# Supplementary figures and images for: Conserved molecular signatures of hygrosensory neurons in two dipteran species
Source: PLoS One. 2026 Apr 28;21(4):e0347993. doi: 10.1371/journal.pone.0347993 (PMC13123972; doi:10.1371/journal.pone.0347993)

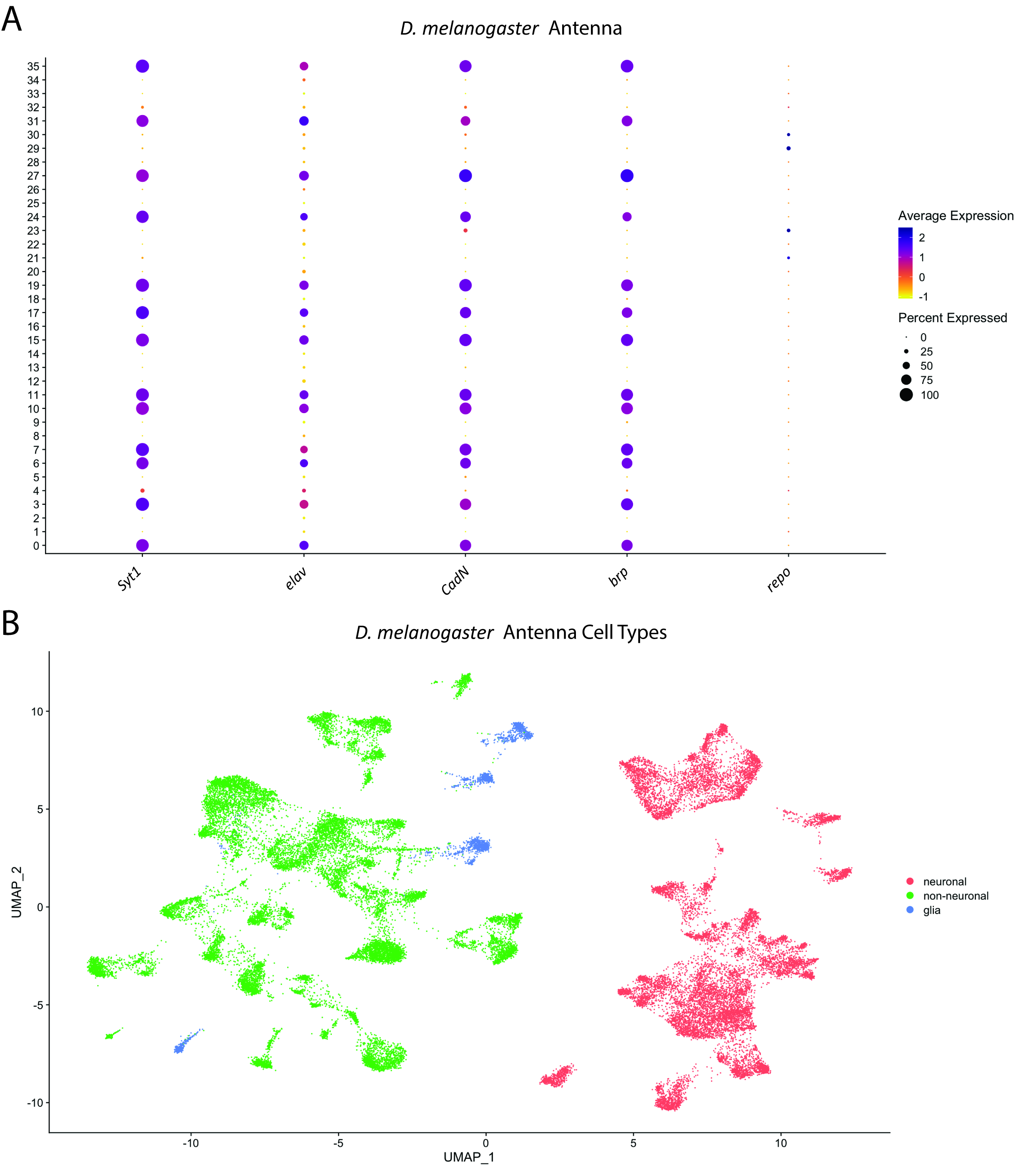

Supplement: S1 Fig — (A) Dot plot showing expression of the neuronal markers Syt1, elav, CadN and brp, and the glial marker repo, across all antennal clusters. (B) UMAP projection of cluster identities assigned as neuronal, non-neuronal or glia based on marker expression. Dot size represents the proportion of nuclei expressing the gene (percent expressed); colour indicates average expression level, ranging from low (yellow) to high (purple). (TIF) [file pone.0347993.s001.tif]

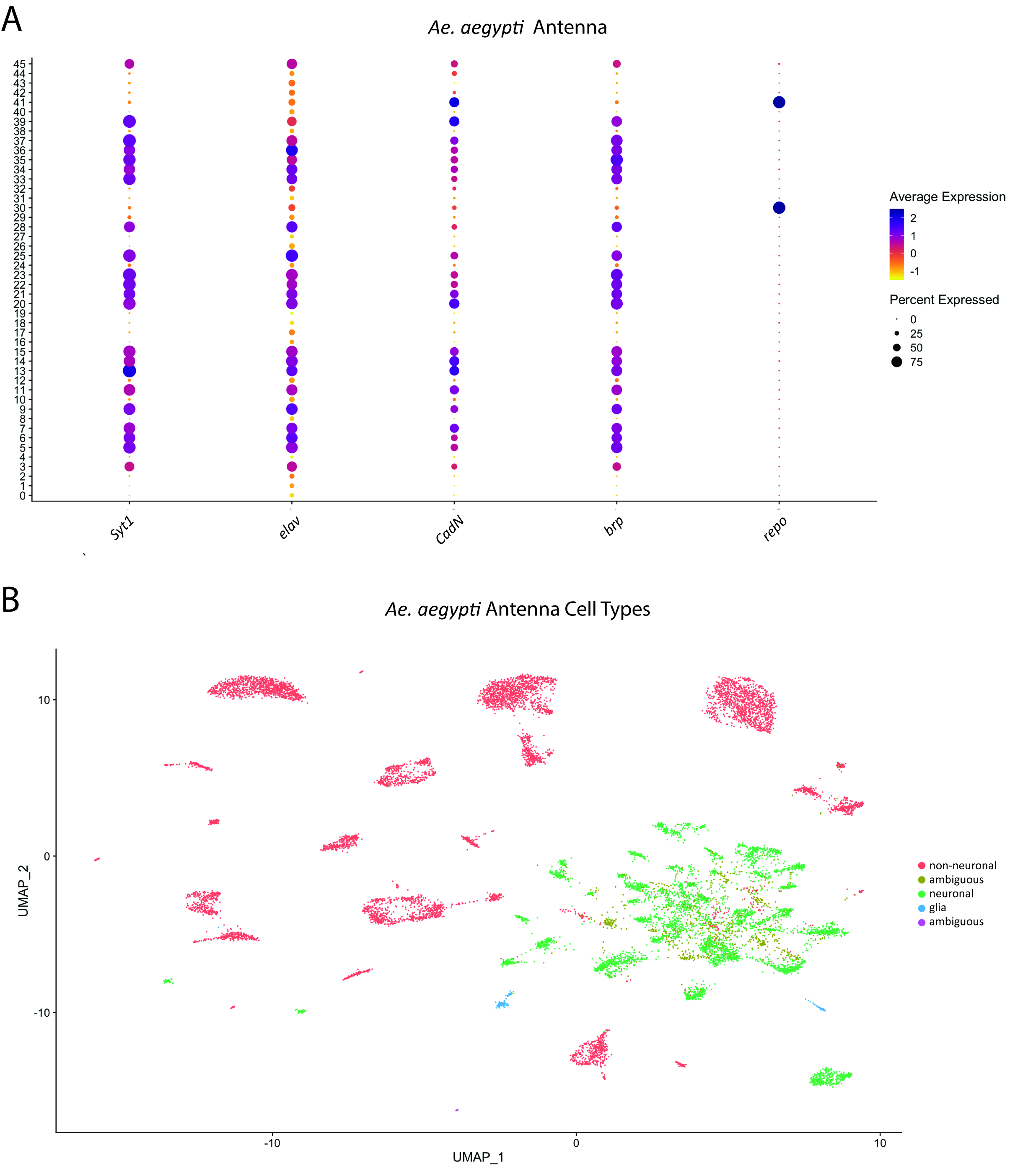

Supplement: S2 Fig — (A) Dot plot showing expression of the neuronal markers LOC5565901 (orthologue to Syt1), LOC5570204 (orthologue to elav), LOC5564848 (orthologue to CadN) and LOC5570381 (orthologue to brp), and the glial marker LOC110678282 (orthologue to repo), across all antennal clusters. (B) UMAP projection of assigned cluster identities. Clusters 3 and 45 displayed ambiguous neuronal marker expression and were classified as ambiguous; all other clusters were assigned as neuronal, non-neuronal or glia. Dot size represents the proportion of nuclei expressing the gene (percent expressed); colour indicates average expression level, ranging from low (yellow) to high (purple). (TIF) [file pone.0347993.s002.tif]

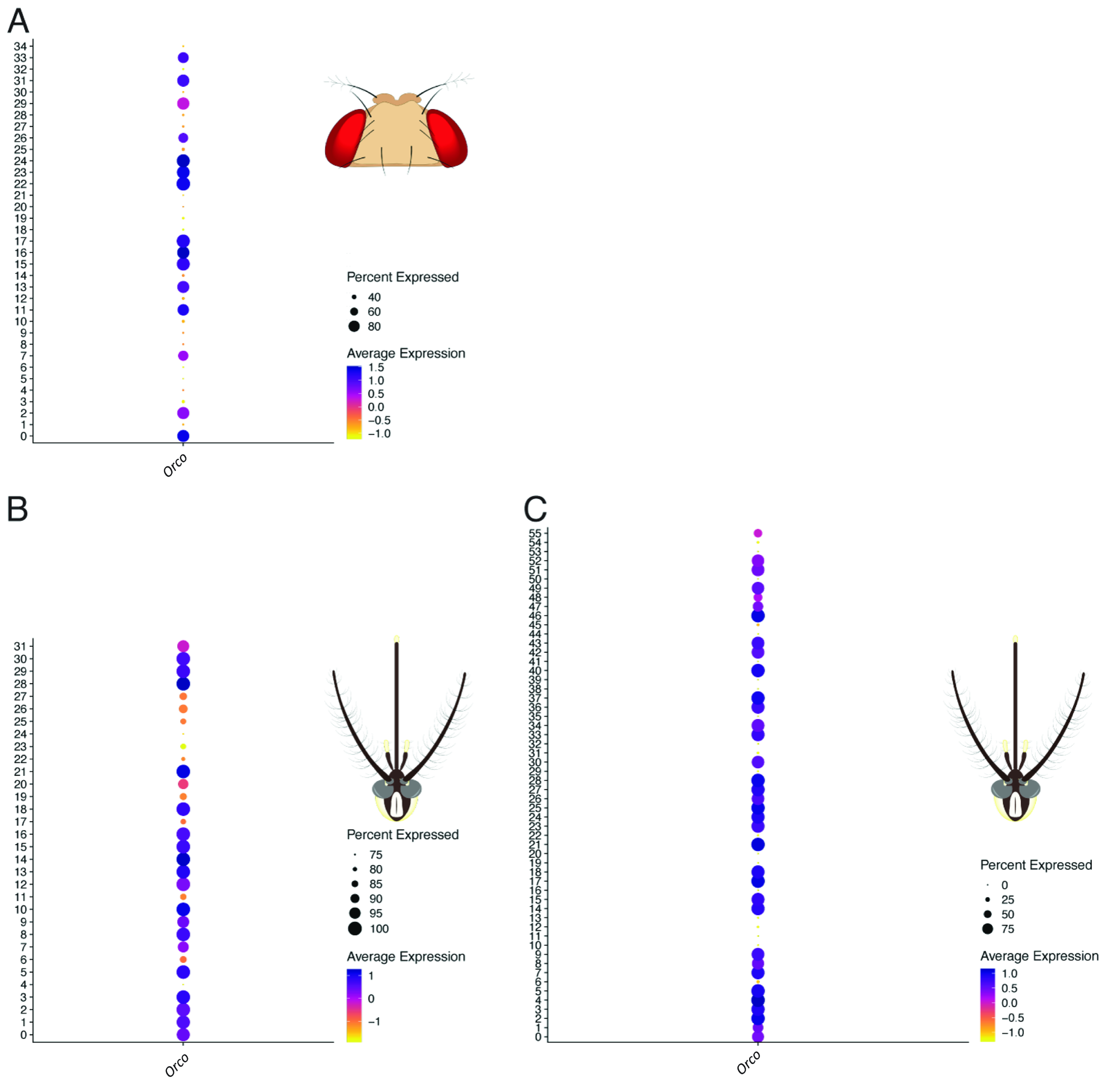

Supplement: S4 Fig — Dot plots showing expression of Orco across all neuronal clusters in (A) D. melanogaster ((B) Ae. aegypti Herre et al. dataset and (C) Ae. aegypti Adavi et al. dataset. In all three datasets, the candidate HRN clusters (clusters 14 and 27 in D. melanogaster; cluster 24 in the Herre et al. dataset; clusters 39 and 41 in the Adavi et al. dataset) show negligible Orco expression, consistent with a hygrosensory rather than olfactory identity. Dot size represents the proportion of nuclei expressing the gene (percent expressed); colour indicates average expression level. (TIF) [file pone.0347993.s004.tif]

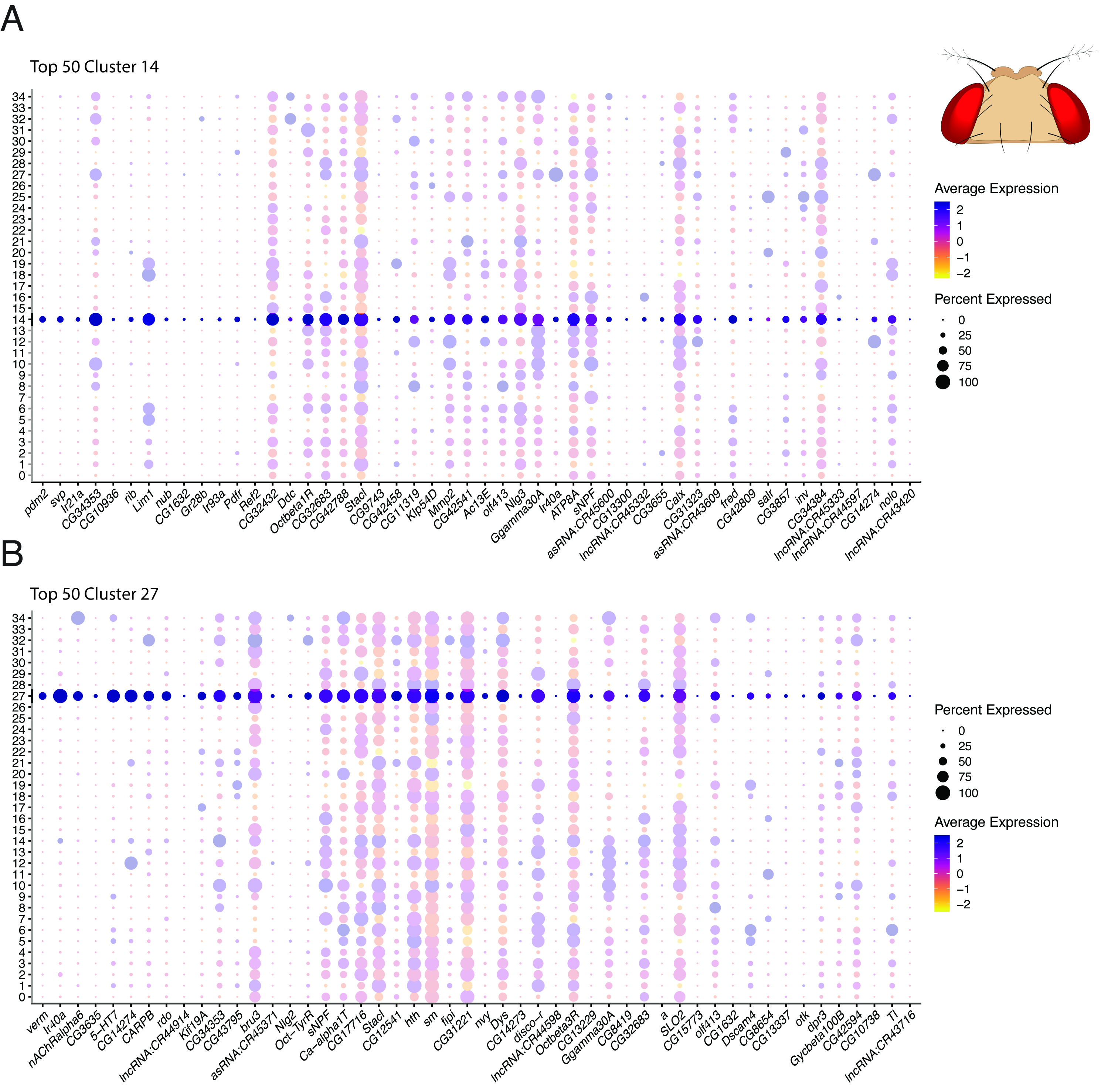

Supplement: S5 Fig — Dot plots showing the top 50 marker genes identified by Seurat FindMarkers for (A) cluster 14 and (B) cluster 27 across all D. melanogaster antennal neuronal clusters. Each panel is displayed across two rows for readability. These marker gene lists were used as input for the cross-species conserved gene analysis. Dot size represents the proportion of nuclei expressing the gene within that cluster (percent expressed); colour indicates average expression level, ranging from low (yellow) to high (purple). (TIF) [file pone.0347993.s005.tif]
